# Automated Processing and Phenotype Extraction of Ovine Medical Images Using a Combined Generative Adversarial Network and Computer Vision Pipeline

**DOI:** 10.3390/s21217268

**Published:** 2021-10-31

**Authors:** James Francis Robson, Scott John Denholm, Mike Coffey

**Affiliations:** Scotland’s Rural College (SRUC), Animal and Veterinary Sciences, Peter Wilson Building, Kings Buildings, West Mains Road, Edinburgh EH9 3JG, UK; scott.denholm@sruc.ac.uk (S.J.D.); mike.coffey@sruc.ac.uk (M.C.)

**Keywords:** generative adversarial network, machine learning, automated medical image processing, deep neural network, animal science, CT scans, computer vision

## Abstract

The speed and accuracy of phenotype detection from medical images are some of the most important qualities needed for any informed and timely response such as early detection of cancer or detection of desirable phenotypes for animal breeding. To improve both these qualities, the world is leveraging artificial intelligence and machine learning against this challenge. Most recently, deep learning has successfully been applied to the medical field to improve detection accuracies and speed for conditions including cancer and COVID-19. In this study, we applied deep neural networks, in the form of a generative adversarial network (GAN), to perform image-to-image processing steps needed for ovine phenotype analysis from CT scans of sheep. Key phenotypes such as gigot geometry and tissue distribution were determined using a computer vision (CV) pipeline. The results of the image processing using a trained GAN are strikingly similar (a similarity index of 98%) when used on unseen test images. The combined GAN-CV pipeline was able to process and determine the phenotypes at a speed of 0.11 s per medical image compared to approximately 30 min for manual processing. We hope this pipeline represents the first step towards automated phenotype extraction for ovine genetic breeding programmes.

## 1. Introduction

Increase in global food demand has led to livestock breeders seeking to produce breeding lines more able to match economic demand which have genetic advantages to primary traits such as growth speed and reduced feed intake. With agricultural animals providing 18% of global calories and 39% of global protein intake, they are still an essential part of global nutritional requirements [1]. One of the methods in making livestock more advantageous is to selectively breed them for commercial traits such as growth rate [2], milk quality [3], weather [4] and disease resistance [5]. Recent improvements in genomic technologies such as detection of single nucleotide polymorphisms (SNPs) and whole genome sequencing [6] have allowed unparalleled insight into the driving factors which guide animal phenotypes [7] and successful genomic breeding selection has been able to identify traits which are not only desirably economically, such as improved livestock social behaviour and carcass composition [5], but also identify novel cosmetic or welfare indicators such as predicting horn phenotypes in Merino sheep [8]. As the number and biological complexity of known phenotypes are increasing, there is a call to innovate new ways to detect phenotypes faster and more accurately [9] in addition to detecting and preserving those of potential future relevance [10].

Non-invasive imaging techniques, such as computed tomography (CT), magnetic resonance imaging (MRI) and ultrasound, can provide detailed data from which phenotypes can then be extracted [11,12] and used in breeding programmes. One major benefit of using these non-invasive imaging techniques is that internal phenotypic data, such as muscle and fat distribution [13], organ size and limb morphology, can then be incorporated more swiftly into genetic breeding programmes for live breeding animals [14]. Out of the commonly used non-invasive imaging techniques, CT scanning provides the highest resolution (1–2 mm). One hurdle which can impact extraction of useful phenotypic information is the processing and analysis of these images which can be time consuming and therefore costly, especially if there is a need to re-analyse historic databases to measure newly emerging phenotypes.

Machine learning and artificial intelligence have been successfully implemented to increase phenotype detection speed and accuracy within many different medical areas including brain cancer detection, COVID status in lungs and classification of organ deformities [15,16,17]. Recently the same technology has been applied to areas of agricultural science such as detection of bovine tuberculosis status based upon milk spectral data [18,19]. Briefly put, these networks work by passing data such as images, or segments thereof, through a series of layers containing artificial neurones which determine the likelihood of visually similar animals such as pigs, sheep or horses on a scale of 0 (absent) to 1 (present). The type of network commonly used to perform this image to binary diagnostic is a convolutional neural network where, as the layer depth increases, many datapoints (such as pixels) are condensed into fewer datapoints (likelihood of, e.g., pig, sheep or horse presence). The subject field of artificial intelligence, machine learning and deep learning using neural networks is extremely broad, and this research article only aims to provide a broad overview in order to demonstrate its application in agriculture and to not discuss these in depth, although many excellent reviews exist for further reading [20,21,22,23].

**Figure 1:**
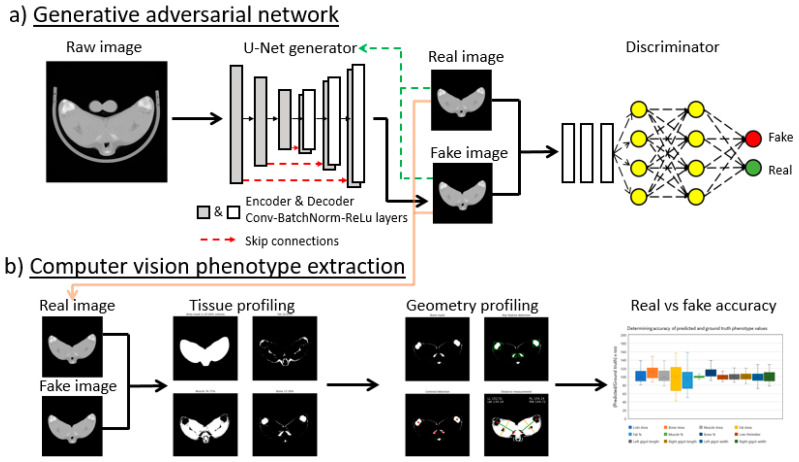
Combined GAN-CV pipeline for phenotype extraction. Neural networks can be trained to perform image-to-image translations such as in (**a**) where a raw ovine CT scan is passed through a generator network, a series of convolution, batch normalisation and ReLu activation function layers, to produce a “fake” image. Skip connections apply regions from the encoded to the encoded images and improve object border definition. By reducing differences between the real and the fake images (green dashes) the autoencoder also learns to better produce fake images independently. A second neural network, the discriminator, then determines if an image is considered real or fake. By pairing the two neural networks to work against each other, an adversarial component emerges, where the generator tries to produces images to fool the discriminator and the discriminator tries to determine if these images are real or not. Phenotype extraction is performed on both real and fake images (**b**) to determine tissue composition and shape before being compared to confirm accuracy.

To perform image-to-image translations a similar type of neural network is required, although rather than condensing pixel information into a few datapoints, the shape of the layers more closely resembles that of an hourglass laying on its side (Figure 1a). This hourglass shape allows the network to perform general purpose image-to-image translation and even increase resolution of blurry input images [24]. By pairing this image-to-image transforming network with a second convolutional neural network (Figure 1a), the discriminator, which compares and scores the images produced by the image transforming network and tries to discriminate between fake and ground truth results, a self-training system can be produced. These two-component image translational networks are termed generative adversarial networks (GANs) and have traditionally been used for a variety of image translational tasks including sketch-to-photo, smile-to-frown, and non-bearded-to-bearded [24,25,26]. More recently, GANs have been applied to medical images to remove noise from low-dose CT, generate tissue structure from blood vessel networks, correct motion artefacts, produce CT images from MR images and synthesise new image data [27,28,29].

By combining GANs with another machine learning technique, computer vision (CV), any images generated by the GAN can then be analysed to extract data of interest in a fully automated way (Figure 1b). Computer vison is a research field which aims to extract understanding or context from images and can use both traditional mathematical regression techniques as well as deep learning classification networks [30,31]. Application of CV can range from simple inspection of food quality and ripeness by counting the number of pixels within images of fruit and vegetables which fall within certain colour hue ranges [32,33] up to more complex tasks such as identifying road signage or pedestrians to guide automated driving systems [34].

We use both smart techniques (GANs and CV, Figure 1a,b, respectively) to aid processing and analysis of agricultural medical images of sheep. This research aims to first implement a GAN to perform ovine CT processing steps involving global information manipulation such as object and organ removal since within the image are multiple objects (scanning cradle and padding) and organs (testes) of varying size, morphology and orientation. Then, with the processed image containing only key features, attempt to extract phenotypes relevant for breeding programmes using CV techniques in an automated process.

## 2. Materials and Methods

### 2.1. Ovine Ischium Scan Collection

A single cross-sectional 2D image was taken through the top of the leg at the point of the ischium for each lamb using a Somatom Scope (Siemens, located at the SRUC-BioSS CT unit in Edinburgh, Scotland) with a slice thickness of 10 mm for a variety of breeds including Beltex, Blue Texel, Charollais, Hampshire Down, Meatlinc, Shropshire, Southdown, Suffolk and Texel as performed by Bunger et al. [12]. The images at this stage are referred to as “raw” images throughout the paper as they are unprocessed. All CT images produced from the scans are exported in the “Digital Imaging and Communications in Medicine” (DICOM) format, a unified filetype for medical imaging techniques. Such DICOM images contain additional data regarding the subject, such as age, sex and location, in addition to collection parameters such as equipment and scanning methodology used. Image dimensions used for this study were 512 × 512 pixels of an intensity value between 0 (black) and 2550 (white) where 0 typically represents low-density matter such as air and 2550 represents extremely dense matter such as metal.

### 2.2. Determination of Tissue Pixel Intensities

Pixel intensities corresponding to respective tissues of fat, muscle and bone were calculated based upon comparison with dissected tissue as explored by Bunger et al. [12]. Pixel intensity windows for fat, muscle and bone were 800–1000, 1000–1100 and 1100–1750, respectively. This group’s previous research allowed us to incorporate set pixel intensity windows for each tissue type into the CV pipeline easily.

### 2.3. Manual Image Processing and Phenotype Analysis

All images had been previously labelled by manual phenotype extraction. Parts of the image superfluous for downstream phenotype determination including scanning cradle and testes were removed using STAR software routines [12] using the method described by Glasbey et al. [35]. Images produced from this processing are considered as “ground truth”. From the ground truth images, tissue phenotype could then be extracted by calculating tissue distribution within the experimentally determined windows. Other phenotypes such as gigot length were measured manually by measuring the distance from the centre of the ischium bone cross-section to that of the femur bone cross-section in a “click and drag” fashion. Processing the images in this fashion took approximately 30 min.

### 2.4. GAN Model

GANs are two-component systems which have a generator component *G* to generate images and a discriminator component *D* to determine if the image is real or fake. The generator *G* takes an input image to translate into an output image *y* and can operate in either an unconditional fashion where random noise *z* is supplied or in a conditional fashion where an input image *x* or random noise *z* is supplied, *G*: {*x* or *z*} → *y.* The discriminator *D* determines if the image produced is “real” or “fake” and helps train the generator *G* to produce images which can pass as “real”. GANs thus attempt to optimize the following function [36]:

(1) $$\begin{array}{cc} \begin{array}{c} \min\\ G \end{array}\begin{array}{c} \max\\ D \end{array}V\left(G,D\right)= & E_{x,y}\;[\log\;D(x,\;y)]+\\ & E_{x,z}\;[\log\;(1-D(x,\;G(x,z))] \end{array}$$

Further improvement of the generator *G* can be incorporated by including a function to minimise the absolute pixel differences between “real” and “fake” images [25].

(2) $$\begin{array}{c} \min\\ G \end{array}L_{L1}\left(G\right)=E_{x,y,z}\left[y-G\left(x,z\right)\right]\;$$

Which results in the following final model:

(3) $$G*=\;\begin{array}{c} \min\\ G \end{array}\begin{array}{c} \max\\ D \end{array}V\left(G,D\right)+L_{L1}\left(G\right)$$

#### 2.4.1. GAN Training

The GAN network trained in this study is an implementation of AUTOMAP [37] and Pix2Pix [25] which has been optimised for use with paired image datasets [38]. This particular GAN was chosen for this study as it was designed from the ground up to process paired sets of images, such as those commonly found in the medical field where an image can be altered to produce a “before” and “after” whilst maintaining the same subject ID and type, e.g., sheep–sheep, human–human, in a conditional synthesis process. This is in contrast to other popular GANs, such as CycleGAN and DCGAN, which perform unconditional synthesis by capturing key style concepts, from large batches of example images to translate images between two highly different abstract style concepts such as horse-to-zebra, photograph-to-Van Gogh or sketch-to-cat [39,40].

A dataset containing 126 raw and ground truth image pairs of mixed breed ovine CT scans taken from 2019–2020 were used for GAN training (Supplementary File S1). DICOM pairs were first split into training (n = 101) and validation (n = 25) datasets (80% and 20%, respectively). The raw and ground truth pairs of DICOM filename IDs were first given a suffix of “_0” or “_1”, respectively, to act as identifiers. All file extensions were then modified to ensure compatibility with the DICOM processing libraries used in this study. The script used to train the GAN, along with the full list of GAN settings used for this study, is available within Supplementary File S2. Key settings for training the GAN were as follows: random translation = 0, epochs = 100, weight for L1 reconstruction loss = 0, weight for L2 reconstruction loss = 10.0, weight for softmax focal reconstruction loss = 1.0, weight for total variation = 10^−3^. Following training, both the L1 (absolute pixel difference) and L2 (mean squared error) were approaching stable values (Supplementary Figure S1).

#### 2.4.2. Image Processing Using Trained GAN on Unseen Data

Thirty-two raw CT scans (Supplementary File S3) taken from 2018–2019 and belonging to the breed Charollais were passed through the trained GAN model to produce “predicted” images that were given a suffix of “_2” to clearly differentiate between the raw and ground truth counterparts (Supplementary File S2).

### 2.5. CT Scan Similarity Comparison

#### 2.5.1. CT Scan Histogram Comparison

**Figure 2:**
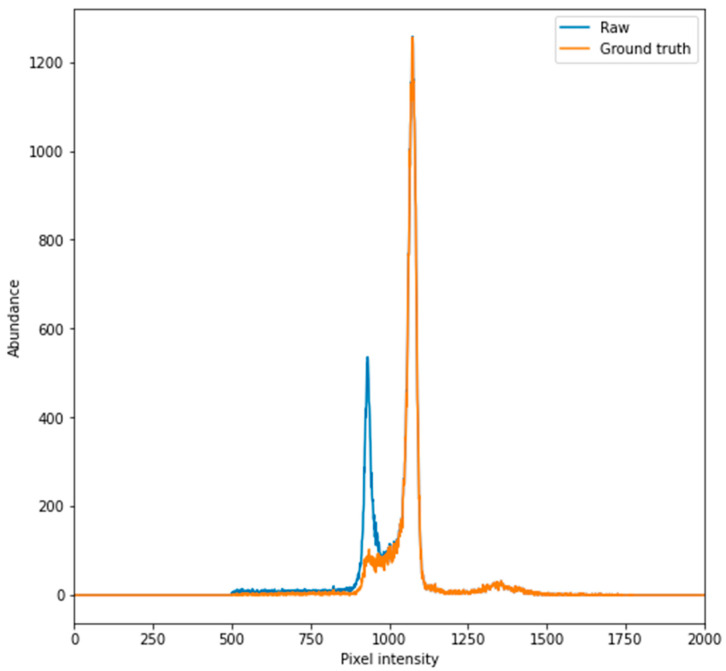
A representative pixel intensity histogram of raw and ground truth image shows large variance. By comparing the raw and ground truth pixel intensity histograms it can be visualised that they a) share certain areas of similarity (as seen at the peak between 1000 and 1250) but also b) contain regions which have different non-zero abundances (within the peak between 750 and 1000). As there are no regions where pixel intensity is either present or not present, images cannot be processed by simply flattening pixel intensities which lie between certain values. This type of non-linear transformation is a task in which neural networks perform well.

Alternative image manipulation techniques, such as removing pixels above or below certain intensities, were not suitable for processing the CT scans in the DICOM format as the pixel intensities of image objects needing to be removed overlapped with that of the subject’s tissue. Furthermore, pixels in certain areas could not be removed since subject orientation was not constant. Due to the large irregular pixel area changes needed to process the images, a deep neural network that can perform image-to-image translations was deemed to be of potential use. This can be visualised by comparing the pixel intensity histograms of both the raw and ground truth images below in Figure 2 (generated as part of the computer vision pipeline in Supplementary File S4).

#### 2.5.2. Calculation of Image Similarity

Mean squared error (MSE) and structural similarity index (SSI) metrics were used to compare the raw and ground truth images with the resulting predicted images. Mean squared error is a full pixel-wise reference metric with values closer to zero being better; it is the sum of the accumulative mean squared difference across each pixel location between a pair of images. This technique, however, is extremely sensitive and seemingly large amounts of MSE can be accumulated by very minor shifts in the image, as perceived by the human eye, such as slight rotations or horizontal and vertical translations [41]. A newer, more holistic and subtle approach which avoids the extreme position sensitivity of MSE is calculating the SSI, which analyses local similarities in structure, luminance and contrast to more closely mimic how the human eye perceives similar images [42]. Both MSE and SSI were calculated for each pairwise comparison of image classes (raw, ground truth or predicted in this study) using the SciKit Image python image processing library as documented in Supplementary File S4 [43].

### 2.6. Phenotype Measurement Using Computer Vision

Automated phenotype extraction from ground truth and predicted (processed) images was performed using a pipeline which incorporated known pixel intensity value thresholding for each component of the carcass, based upon manual dissection, for each tissue type in combination. Geometric phenotypes were computed predominantly using the area, contour and perimeter functions within the CV library SciKit Image [43]. In addition, a set of bespoke functions were also written to detect probable tissue pixel intensity windows of fat, muscle and bone if no known set values were available, or if the images being analysed were from different sources. All steps of phenotype extraction using computer vision are documented in Supplementary File S4.

#### 2.6.1. Tissue Distribution

The areas of all tissues within the ground truth and predicted images were calculated using the SciKit Image contour function for later use in determining percentage tissue composition. Tissue masks for each image were applied by first setting pixel intensity values (fat, muscle, bone) outside the respective tissue windows to zero and then setting values within the window to max (2550). Fat, muscle and bone % of each image were determined by comparing the number of pixels that fell within each of the respective tissue masks to that of the area of all tissue. By visualising each of the tissue masks independently, muscle and fat distribution could be observed in addition to locations of key physical features such as bones for further geometric phenotype analysis.

#### 2.6.2. Skeleton Geometry

One key phenotype used for estimation of muscularity is the ratio of the length and width of the gigot muscle. These dimensions are typically measured by hand from the CT scan image but, by using CV models, we can extract this information automatically from the bone tissue mask image by implementing SciKit Image area and crofton perimeter functions [44]. Since small pieces of grit and sand may appear in the bone mask, due to high density as detected by X-rays, only bone mask objects over 200 pixels in both area and perimeter are referenced. Then, to avoid including spinal bone tissue, the four largest objects in the most +Y direction are assumed to be the features of interest and are placed into pairs according to their position along the X axis. The distance in pixels is then calculated between each pair of bones to determine gigot length. A line perpendicular to that between the bone pairs is then used to find the furthest non-zero positions within the muscle tissue mask and thus determine gigot width.

### 2.7. Computing Hardware and Software

The training of machine learning models can be an intensive computational task which typically requires powerful graphics processing units (GPUs). As such, all computation was performed on an NVIDIA DGX Station workgroup server [45]. The DGX workstation provided supercomputing performance with one out of a total of four TESLA V100 GPUs being used for computations underpinned by an Ubuntu operating system. All code was run within a Compute Unified Device Architecture (CUDA) 10.1 docker container which allows parallelisation of general-purpose processing to be applied to the powerful GPUs. Within this container, the open source learning framework Chainer was used to accelerate creation of the neural networks [46]. The GAN trained in this study is an implementation of AUTOMAP [37] and Pix2Pix [25] which has been optimised for use with paired image datasets [38]. Predicted images produced by the GAN were then processed using a bespoke python script run within a Jupyter notebook (Supplementary File S4). The notebook contains code within cells which can either (a) run individual steps and generate intermediary output figures (slower) or (b) calculate metrics and compare images without visualising any medical images (faster).

## 3. Results

The trained model was able to transform the raw images with a high degree of accuracy and perform the large image area manipulations, such as scanning cradle and testicle removal, needed to produce images similar to the manually processed ground truth images. The accuracy of these transformations was confirmed by visual inspection of predicted images and measurement of image similarity metrics including MSE and SSI. Phenotypic traits such as fat, muscle and bone tissue distribution and both gigot length and width were then automatically extracted from the predicted (transformed) images using CV techniques. All values calculated using this pipeline area are recorded in an output file (Supplementary File S5).

### 3.1. CT Scan Processing Using Trained GAN

**Figure 3:**
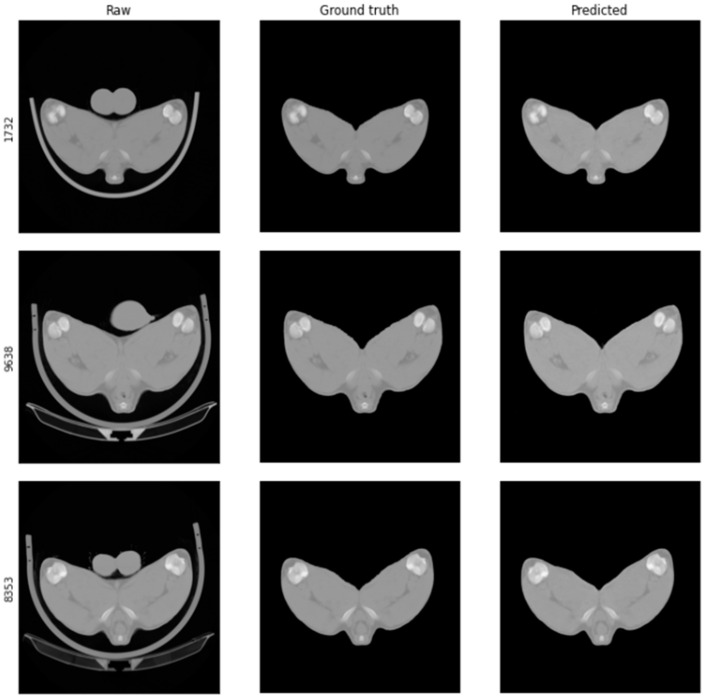
Representative comparison of raw, ground truth and predicted CT scan images. A trained generative adversarial network (GAN) was used to process raw CT images (**left** column) into something resembling manually processed ground truth images (**middle** column). Non-quantitative visual inspection of predicted results (**right** column) indicated that images produced by this GAN are similar to ground truth counterparts. The GAN showed good capabilities in automatically handling the large image transformations needed to remove image objects such as testes and scanning cradle.

**Figure 4:**
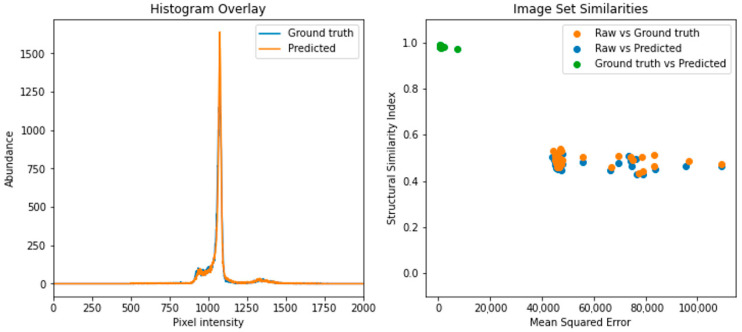
Quantifying a high degree of quantified similarity between ground truth and predicted images. Comparing a representative pixel intensity histogram of a ground truth and predicted image (**left**) showed a high degree of overlay and that peaks were present in similar areas at similar amplitudes, indicating a similar distribution of pixel intensities within each image. Structural components of image groups were compared (**right**) using mean squared error (MSE) and structural similarity indexes (SSIs) which revealed a) high average MSE (58,674 ± 17,766 and 58,008 ± 17,319, n = 32) with low average SSI (0.49 ± 0.025 and 0.48 ± 0.024, n = 32) between raw vs. ground truth and raw vs. predicted image groups, respectively, b) low average MSE (1028 ± 1201) and high average SSI (0.98 ± 0.0035) when comparing ground truth vs. predicted images. These high SSI and low MSE values confirm the suitability of a trained generative adversarial network to perform highly accurate ovine CT image processing.

Raw CT scans not previously seen by the GAN were processed using the trained model at a speed of 0.11 s per scan. Predicted and ground truth images and pixel intensity histograms were first compared visually to initially assess GAN suitability and ensure that they were visually similar (Figure 3). Quantitative metrics such as MSE and SSI were further determined to accurately assess the success of the GAN for processing the CT scans (Figure 4).

#### 3.1.1. Images Produced from Trained Model

The trained model was able to perform the major structural alterations within the image dataset needed to transform the raw CT scans into something which, by eye, strongly resembled the ground truth images as shown below in Figure 3. Image IDs 1732, 9638 and 8353 were chosen to illustrate this transformation since, on visual inspection, they contained the largest area of features needing to be removed (large testes and a large scanning cradle).

#### 3.1.2. Image Similarity Metrics Confirm a High Degree of Similarity

Just as raw and ground truth image histograms were compared previously, likewise the ground truth and predicted images were compared in a similar fashion which revealed two histograms, highly similar, showing a large proportion of overlap and a high degree of similarity from visual inspection. The likeness of the raw, ground truth and predicted image sets (n= 32) was compared pairwise using MSE and SSI. Both raw vs. ground truth and raw vs. predicted showed the lowest image similarity values with an average MSE of 58,674 ± 17,766 and 58,008 ± 17,319 and with average SSIs of 0.49 ± 0.025 and 0.48 ± 0.024, respectively, indicating a high degree of image dissimilarity. On the other hand, comparing images in the ground truth and predicted datasets showed a much lower average MSE (1028 ± 1201) and a far higher average SSI of 0.98 ± 0.0035, indicating a far greater similarity and indicating high accuracy of the trained model in mimicking the manual processing of CT scan images.

### 3.2. Automated Phenotype Extraction

The image processing library SciKit Image was successfully implemented to provide CV capabilities in the automated phenotype extraction pipeline. In this study, phenotypes of interest included fat, muscle and bone tissue abundance as well as leg geometry such as length and width (Supplementary File S4).

#### 3.2.1. Leg Tissue Composition

**Figure 5:**
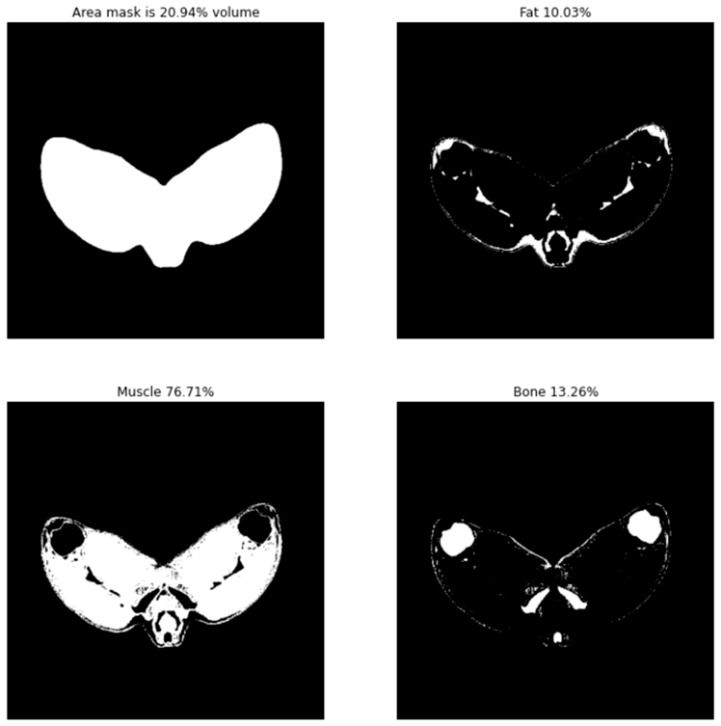
Representative tissue distribution of fat, muscle and bone within tissue area of predicted images. The results of the trained generative adversarial network were analysed by examining total area (**top left**) and by applying pixel intensity threshold windows to separately visualise fat (**top right**), muscle (**bottom left**) and bone (**bottom right**). The total number of pixels that fell within these pixel intensity windows determined the volume of the respective tissue types in the sample since 1 pixel = 1 mm^2^.

**Figure 6:**
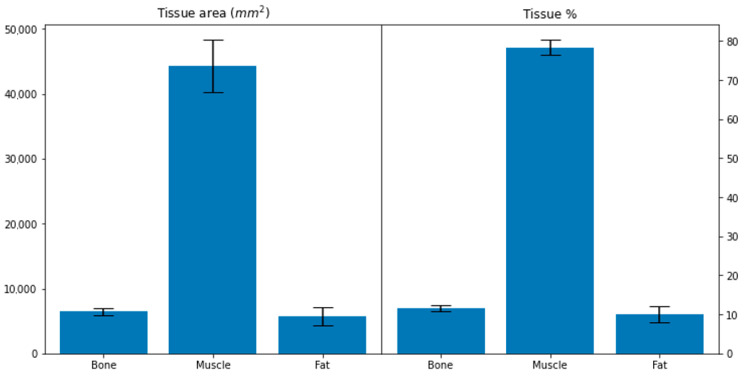
Area and percentage composition of tissue types within predicted ovine medical images. Using the threshold windows for each of the respective tissue types, the total area occupied was calculated for each tissue type (**left**) and what percentage this represented within each individual CT scan (**right**). On average, the area of bone, muscle and fat across the dataset was 6488 ± 533, 44,274 ± 4051, 5712 ± 1377. Carcass tissue composition percentage-wise for bone, muscle and fat was 11.52 ± 0.78, 78.41 ± 1.90 and 10.07 ± 2.03%.

Tissue abundance and distribution of fat, muscle and bone, within the single 2D image analysed, were calculated by counting pixels which fell within experimentally determined tissue pixel intensity windows compared to the total tissue area. Binary visualisation of these tissue value windows allowed rapid profiling of tissue distribution as seen below in Figure 5. Using this method, tissue abundances were calculated for each medical image in terms of both area and percentage composition (Figure 6). On average, the area of bone, muscle and fat across the dataset was 6488 ± 533, 44,274 ± 4051 and 5712 ± 1377 mm^2^. Carcass tissue composition percentage-wise for bone, muscle and fat was 11.52 ± 0.78, 78.41 ± 1.90 and 10.07 ± 2.03%.

#### 3.2.2. Gigot Length and Width Phenotype Extraction

**Figure 7:**
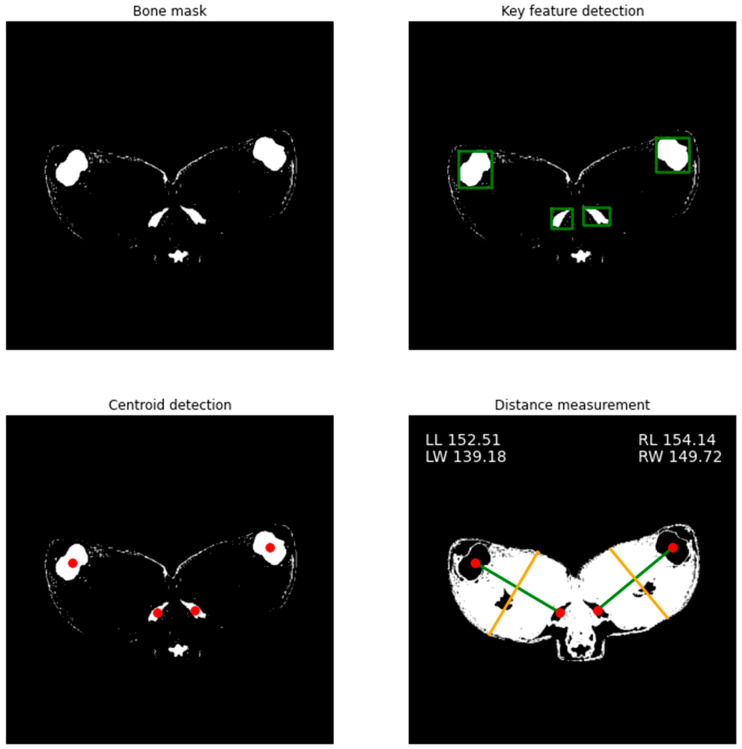
Automated identification of key features and determination of gigot length and width from predicted images. Using the bone tissue mask (**top left**) from the predicted image generated by the GAN, key features can be identified (**top right**). The centroid of each object within each feature area can be calculated (**bottom left**) to then measure distances and determine gigot length (LL, RL **bottom right**). By taking the perpendicular equation of the line which connects the two pairs of bones, the width of the gigot (LW, RW, **bottom right**) can be calculated by discovering the first and last non-zero values of these positions within the muscle tissue mask. Left and right gigot lengths on average were 164.45 ± 8.72 mm and 166.38 ± 9.71 mm with widths being 137.55 ± 10.53 mm and 143.99 ± 12.42, respectively.

By applying CV functions from the SciKit Image library such as area, perimeter and location restraints to objects in the bone tissue mask, the position and centre of key features were detected, and gigot length and width determined automatically as part of the CV script (Supplementary File S4). This process is visualised below in Figure 7. Left and right gigot lengths were 164.45 ± 8.72 mm and 166.38 ± 9.71 mm with widths being 137.55 ± 10.53 mm and 143.99 ± 12.42, respectively.

#### 3.2.3. Phenotype Extraction Accuracy

**Figure 8:**
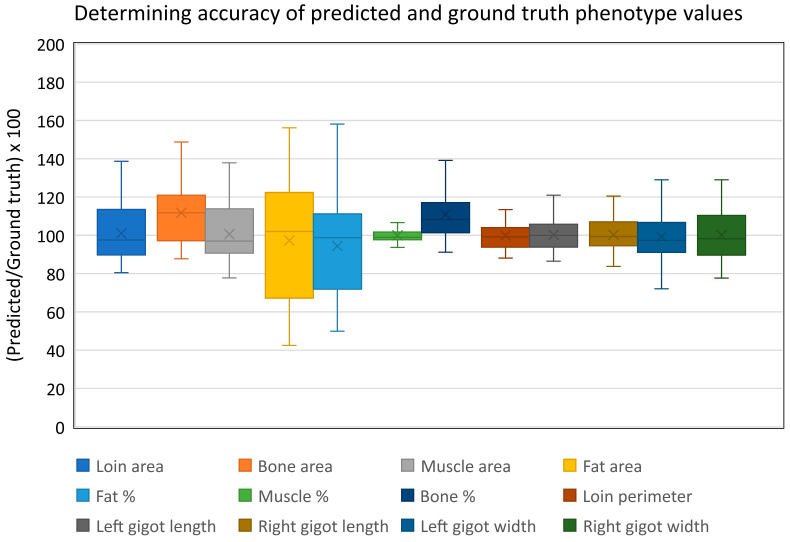
Comparing values of predicted and ground truth phenotypes. Prediction estimation accuracy was determined by comparing phenotype values generated from both predicted and ground truth datasets using the computer vision pipeline. Across all phenotypes, predicted values for each image were on average 101.44% that of the ground truth value with a standard deviation of 12.90% (n = 32).

Phenotypes from both predicted and ground truth datasets were extracted using the computer vision pipeline and compared to determine the suitability of predicted images for phenotype determination as seen below in Figure 8. Across all phenotypes, the average values were on average 101.44% that of the ground truth value with a standard deviation of 12.90% (n = 32). Muscle % was the most accurate predicted phenotype with estimated values between 93.67 and 106.65%. On the other hand, calculated fat area was the least accurate predicted phenotype with estimated values between 42.50 and 156.18% (following incomplete ovine testes removal from image ID 8346, fat-related phenotypes were not included in accuracy calculations as testes are calculated as fatty tissue. All other phenotypes for this image were recorded normally such as muscle area, bone area and gigot geometry).

## 4. Discussion

Continued reduction in DNA genotyping cost over time has resulted in mainstream integration of genomic selection into genetic improvement programmes for a number of domesticated animals. The increase in availability of genotypes leads to the need to identify the correlated phenotypes, as subtle or rare as they may be [10]. One technology which shows great promise in detecting these subtle phenotypes is the use of trained neural networks and CV. The processing and extraction of key data from medical images in the past have been typically performed manually by trained and experienced professionals. However, more recently, emergence of trained artificial intelligence networks has contributed to increased analysis throughput and accuracy of phenotype determination, such as the increased use and accuracy of neural networks for cancer and disease detection compared to the results of medical professionals [15,16,47,48]. By implementing similar techniques in the field of animal breeding, we hope to enhance the speed and accuracy of phenotype detection to streamline swift integration into genetic improvement programmes.

As part of this automated pipeline, a generative adversarial network was first trained to perform the necessary image-to-image translation required for automatically processing previously unseen CT scan images for subsequent phenotype extraction using CV at a speed of 0.11 s per image, this speed is far greater than the approximate 30 min required to manually process the image. The resultant images processed in this manner had an SSI of (0.98 ± 0.0035) when compared to the manually processed ground truths according to their structural similarity index and were visually indistinguishable. Automated phenotype extraction from predicted CT images was then performed by subdividing each image into the respective tissue masks to display the fat, muscle and bone volume and distribution. By using key feature detection within the bone image mask, distances between ischium and femur bone cross-sections were calculated to determine the geometric phenotype of gigot length and width. Phenotype values determined using the computer vision pipeline were on average 101.44% that of the ground truth value with a standard deviation of 12.90% (n = 32), indicating a high level of accuracy across the population.

One of the potential limitations of this study was the small training dataset (m = 126) as development of neural networks typically uses datasets numbering in the thousands. However, this limited dataset did not cause any major issues in accuracy as ground truth and predicted images showed an SSI of 0.98 ± 0.0035 and were indistinguishable by eye. One possible reason for such high accuracy with this limited dataset was that all subjects within the CT scans were constrained to similar postures. This hypothesis was later confirmed by re-introducing artificial random movement (such as rotation or vertical/horizontal shifts) into the images used for GAN training, resulting in a higher validation loss, poorer network performance and blurry resultant images (Supplementary Figures S1–S9).

Unfortunately, using this limited dataset resulted in one of the unseen images containing a small amount of testis tissue following processing with the GAN which was then incorrectly quantified as fat tissue. In the future, as more images are integrated into the model, we believe that the accuracy of the GAN shall improve which shall directly improve the precision of the CV phenotype determination pipeline.

## 5. Conclusions

In summary, we believe this research represents the first case of using an automated phenotype detection pipeline on agricultural animal medical images. This was achieved by using a combined GAN-CV pipeline to analyse agricultural medical images in a fully automated fashion. By feeding a paired image dataset into a GAN, we were able to perform the various image processing steps needed to produce a predicted image, containing only the relevant tissues, with accuracies of 98% which rivalled that of manual processing and at a fraction of the cost. Phenotypes were then extracted or calculated from these predicted images by applying CV techniques as part of an automated pipeline.

We hope to immediately expand this highly accurate GAN-CV pipeline to process and extract phenotypes from other key CT scan sections such as the 8th thoracic vertebra and 5th lumbar vertebra positions. Further on, we hope to develop a pipeline to process a complete set of layered CT images to produce an accurate 3D model from which a multitude of phenotypes can then be extracted, such as spine length and vertebra number, and detect phenotypes which are best explored in 3D space such as organ morphology [49,50]. By continuing this research we will further expand the automated extraction of phenotypes from agricultural medical imaging data and use the findings to guide genetic and genomic breeding programmes.

## Data Availability

All raw and ground truth training images used in this study are included as part of Supplementary File S1. All raw, ground truth and predicted images from the unseen medical images are included as part of Supplementary File S3. The trained model is provided as Supplementary File S6.

## Supplementary Materials

The following are available online at https://www.mdpi.com/article/10.3390/s21217268/s1, Performance of generator and discriminator networks under varying degrees of random translation (Figures S1–S6), Raw, ground truth and predicted images produced from the networks with varying degrees of random translation (Figures S7–S9), Training images (File S1), Script for GAN training (File S2), Unseen images (File S3), Computer vision python notebook (File S4), Results table (File S5), Trained GAN model (File S6).

## References

[1] FAO Shaping the future of livestock; Report No. I8384EN. Proceedings of the 10th Global Forum for Food and Agriculture.

[2] Rexroad C., Vallet J., Matukumalli L.K., Reecy J., Bickhart D., Blackburn H., Boggess M., Cheng H., Clutter A., Cockett N. (2019). Genome to phenome: Improving animal health, production, and well-being—A new USDA blueprint for animal genome research 2018–2027. Front. Genet..

[3] Gonzalez-Recio O., Coffey M.P., Pryce J.E. (2014). On the value of the phenotypes in the genomic era. J. Dairy Sci..

[4] Sánchez-Molano E., Kapsona V.V., Ilska J.J., Desire S., Conington J., Mucha S., Banos G. (2019). Genetic analysis of novel phenotypes for farm animal resilience to weather variability. BMC Genet..

[5] Brito L.F., Oliveira H.R., McConn B.R., Schinckel A.P., Arrazola A., Marchant-Forde J.N., Johnson J.S. (2020). Large-Scale Phenotyping of Livestock Welfare in Commercial Production Systems: A New Frontier in Animal Breeding. Front. Genet..

[6] Li X., Yang J., Shen M., Xie X.L., Liu G.J., Xu Y.X., Lv F.H., Yang H., Yang Y.L., Liu C.B. (2020). Whole-genome resequencing of wild and domestic sheep identifies genes associated with morphological and agronomic traits. Nat. Commun..

[7] Santos B.F.S., Van Der Werf J.H.J., Gibson J.P., Byrne T.J., Amer P.R. (2017). Genetic and economic benefits of selection based on performance recording and genotyping in lower tiers of multi-tiered sheep breeding schemes. Genet. Sel. Evol..

[8] Duijvesteijn N., Bolormaa S., Daetwyler H.D., Van Der Werf J.H.J. (2018). Genomic prediction of the polled and horned phenotypes in Merino sheep. Genet. Sel. Evol..

[9] Seidel A., Krattenmacher N., Thaller G. (2020). Dealing with complexity of new phenotypes in modern dairy cattle breeding. Anim. Front..

[10] Leroy G., Besbes B., Boettcher P., Hoffmann I., Capitan A., Baumung R. (2016). Rare phenotypes in domestic animals: Unique resources for multiple applications. Anim. Genet..

[11] Han D., Lehmann K., Krauss G. (2009). SSO1450—A CAS1 protein from Sulfolobus solfataricus P2 with high affinity for RNA and DNA. FEBS Lett..

[12] Bunger L., Macfarlane J.M., Lambe N.R., Conington J., McLean K.A., Moore K., Glasbey C.A., Simm G. (2011). Use of X-ray Computed Tomography (CT) in UK Sheep Production and Breeding. CT Scanning-Tech. Appl..

[13] Lee S., Lohumi S., Lim H.S., Gotoh T., Cho B.K., Jung S. (2015). Determination of intramuscular fat content in beef using magnetic resonance imaging. J. Fac. Agric. Kyushu Univ..

[14] McLaren A., Kaseja K., McLean K.A., Boon S., Lambe N.R. (2021). Genetic analyses of novel traits derived from CT scanning for implementation in terminal sire sheep breeding programmes. Livest. Sci..

[15] Savage N. (2020). How-ai-is-improving-cancer-diagnost. Nature.

[16] Lin L., Qin L., Xu Z., Yin Y., Wang X., Kong B., Bai J., Lu Y., Fang Z., Song Q. (2020). Using Artificial Intelligence to Detect COVID-19 and Community-acquired Pneumonia Based on Pulmonary CT: Evaluation of the Diagnostic Accuracy. Radiology.

[17] Lim L.J., Tison G.H., Delling F.N. (2020). Artificial Intelligence in Cardiovascular Imaging. Methodist Debakey Cardiovasc. J..

[18] Denholm S.J., Brand W., Mitchell A.P., Wells A.T., Krzyzelewski T., Smith S.L., Wall E., Coffey M.P. (2020). Predicting bovine tuberculosis status of dairy cows from mid-infrared spectral data of milk using deep learning. J. Dairy Sci..

[19] Brand W., Wells A.T., Smith S.L., Denholm S.J., Wall E., Coffey M.P. (2021). Predicting pregnancy status from mid-infrared spectroscopy in dairy cow milk using deep learning. J. Dairy Sci..

[20] Soffer S., Ben-Cohen A., Shimon O., Amitai M.M., Greenspan H., Klang E. (2019). Convolutional Neural Networks for Radiologic Images: A Radiologist’s Guide. Radiology.

[21] Hou X., Gong Y., Liu B., Sun K., Liu J., Xu B., Duan J., Qiu G. (2018). Learning based image transformation using convolutional neural networks. IEEE Access.

[22] Bod M. (2001). A guide to recurrent neural networks and backpropagation. Rnn Dan Bpnn.

[23] Benos L., Tagarakis A.C., Dolias G., Berruto R., Kateris D., Bochtis D. (2021). Machine learning in agriculture: A comprehensive updated review. Sensors.

[24] Parmar N., Vaswani A., Uszkoreit J., Kaiser L., Shazeer N., Ku A., Tran D. Image transformer. Proceedings of the 35th International Conference on Machine Learning ICML 2018.

[25] Isola P., Zhu J.Y., Zhou T., Efros A.A. Image-to-image translation with conditional adversarial networks. Proceedings of the 2017 IEEE Conference on Computer Vision and Pattern Recognition, CVPR 2017.

[26] Zhong G., Gao W., Liu Y., Yang Y., Wang D.H., Huang K. (2020). Generative adversarial networks with decoder–encoder output noises. Neural Networks.

[27] Singh N.K., Raza K. (2020). Medical Image Generation using Generative Adversarial Networks. arXiv.

[28] Armanious K., Jiang C., Fischer M., Küstner T., Hepp T., Nikolaou K., Gatidis S., Yang B. (2020). MedGAN: Medical image translation using GANs. Comput. Med. Imaging Graph..

[29] Frid-Adar M., Diamant I., Klang E., Amitai M., Goldberger J., Greenspan H. (2018). GAN-based synthetic medical image augmentation for increased CNN performance in liver lesion classification. Neurocomputing.

[30] Voulodimos A., Doulamis N., Doulamis A., Protopapadakis E. (2018). Deep Learning for Computer Vision: A Brief Review. Comput. Intell. Neurosci..

[31] Meer P., Mintz D., Rosenfeld A., Kim D.Y. (1991). Robust regression methods for computer vision: A review. Int. J. Comput. Vis..

[32] Wu D., Sun D.W. (2013). Colour measurements by computer vision for food quality control—A review. Trends Food Sci. Technol..

[33] Brosnan T., Sun D.W. (2004). Improving quality inspection of food products by computer vision—A review. J. Food Eng..

[34] Deva Koresh J. (2019). Computer Vision Based Traffic Sign Sensing for Smart Transport. J. Innov. Image Process..

[35] Glasbey C.A., Young M.J. (2002). Maximum a posteriori estimation of image boundaries by dynamic programming. J. R. Stat. Soc. Ser. C Appl. Stat..

[36] Goodfellow I., Pouget-Abadie J., Mirza M., Xu B., Warde-Farley D., Ozair S., Courville A., Bengio Y. (2020). Generative adversarial networks. Commun. ACM.

[37] Zhu B., Liu J.Z., Cauley S.F., Rosen B.R., Rosen M.S. (2018). Image reconstruction by domain-transform manifold learning. Nature.

[38] Kaji S., Kida S. (2019). Overview of image-to-image translation by use of deep neural networks: Denoising, super-resolution, modality conversion, and reconstruction in medical imaging. Radiol. Phys. Technol..

[39] Radford A., Metz L., Chintala S. Unsupervised representation learning with deep convolutional generative adversarial networks. Proceedings of the 4th International Conference on Learning Representations, ICLR 2016.

[40] Zhu J.Y., Park T., Isola P., Efros A.A. (2017). Unpaired Image-to-Image Translation Using Cycle-Consistent Adversarial Networks. Proc. IEEE Int. Conf. Comput. Vis..

[41] Sara U., Akter M., Uddin M.S. (2019). Image Quality Assessment through FSIM, SSIM, MSE and PSNR—A Comparative Study. J. Comput. Commun..

[42] AGandhi S., Kulkarni C.V. (2013). MSE vs. SSIM. Int. J. Sci. Eng. Res..

[43] Van Der Walt S., Schönberger J.L., Nunez-Iglesias J., Boulogne F., Warner J.D., Yager N., Gouillart E., Yu T. (2014). Scikit-image: Image processing in python. PeerJ.

[44] Séverine R. (2011). Analyse D’image Géométrique et Morphométrique par Diagrammes de Forme et Voisinages Adaptatifs Généraux. Ph.D. Thesis.

[45] NVIDIA NVIDIA DGX Station: AI Workstation for Data Science Teams. https://www.nvidia.com/en-gb/data-center/dgx-station-a100/.

[46] Tokui S., Okuta R., Akiba T., Niitani Y., Ogawa T., Saito S., Suzuki S., Uenishi K., Vogel B., Vincent H.Y. Chainer: A deep learning framework for accelerating the research cycle. Proceedings of the 25th ACM SIGKDD International Conference on Knowledge Discovery & Data Mining.

[47] Lassau N., Ammari S., Chouzenoux E., Gortais H., Herent P., Devilder M., Soliman S., Meyrignac O., Talabard M.P., Lamarque J.P. (2021). Integrating deep learning CT-scan model, biological and clinical variables to predict severity of COVID-19 patients. Nat. Commun..

[48] Saood A., Hatem I. (2021). COVID-19 lung CT image segmentation using deep learning methods: U-Net versus SegNet. BMC Med. Imaging.

[49] Nguyen-Phuoc T., Li C., Theis L., Richardt C., Yang Y.L. HoloGAN: Unsupervised learning of 3D representations from natural images. Proceedings of the 2019 International Conference on Computer Vision Workshop, ICCVW 2019.

[50] Öngün C., Temizel A. (2019). Paired 3D model generation with conditional generative adversarial networks. Proceedings of the European Conference on Computer Vision, ECCV 2018 Workshops.

