# Supplementary material for: Automated Processing and Phenotype Extraction of Ovine Medical Images Using a Combined Generative Adversarial Network and Computer Vision Pipeline
Source: Sensors (Basel). 2021 Oct 31;21(21):7268. doi: 10.3390/s21217268 (PMC8588206; doi:10.3390/s21217268)
Supplement: Supplementary file 1 [file sensors-21-07268-s001.zip › BothSupplementary/Supplementary Figures/Supplementary Figures.pdf]

Supplementary Figures

Graphed loss of the discriminator and generator components of the trained GAN.

A GAN is the term which refers to a combination of two or more separate models. Firstly, a generator model performs transformations on an input image. Common metrics which evaluate the generator model performance of how similar this produced images matches the original includes L1 (Absolute difference) or L2 values (Mean squared error). Secondly a discriminator network tries to determine if the produced image is real or fake. Since two networks are being trained at the same time to work against each other, the networks never truly stabilise. For example, a highly performing generator would result in the discriminator performing at 50% accuracy, like a coin flip.

In most GANs, a degree of robustness is built into the image processing, to be able to handle images taken in a similar way but perhaps from different angles or positions. However, for us this had the opposite effect since our ovine medical images were taken from a fixed position in a controlled setting. By pruning part of the GAN which provided this typical robustness, we achieved a higher accuracy and image sharpness. This was provided as a value termed random translation (RT) which we varied between 4 to 0.

Reducing RT from 4 to 2 and then further to 0 resulted in a much more stable network with less oscillation (Figures 1-6). Images produced from the GAN were also less blurry on visual inspection and more closely resembled the target image, by reducing the RT from 2 to 0 this blurring was minimised further (Figures S7-S9).

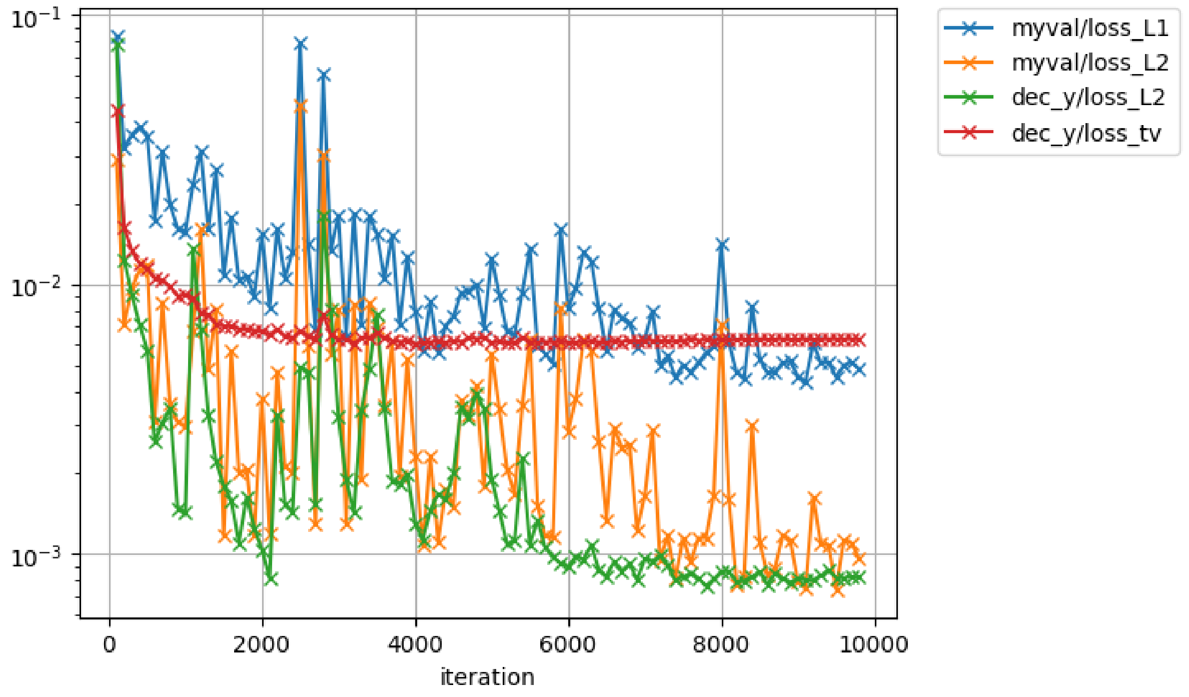

Figure S1. RT4 Generator Loss

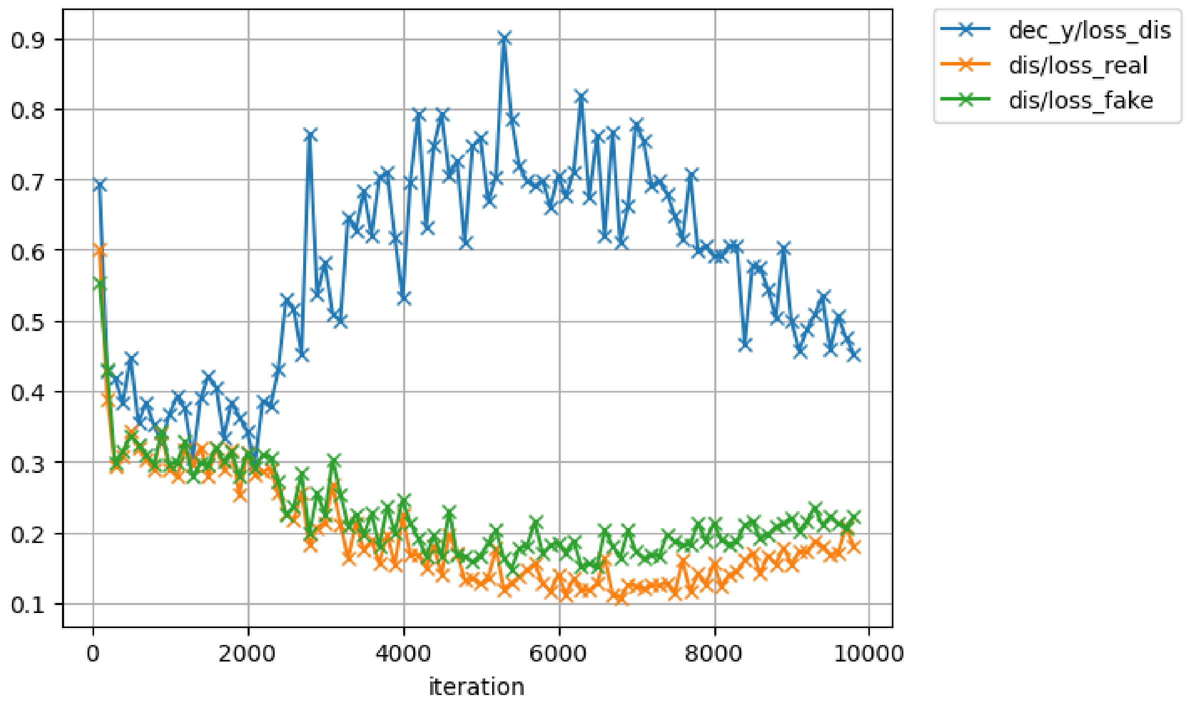

Figure S2. RT4 Discriminator Loss

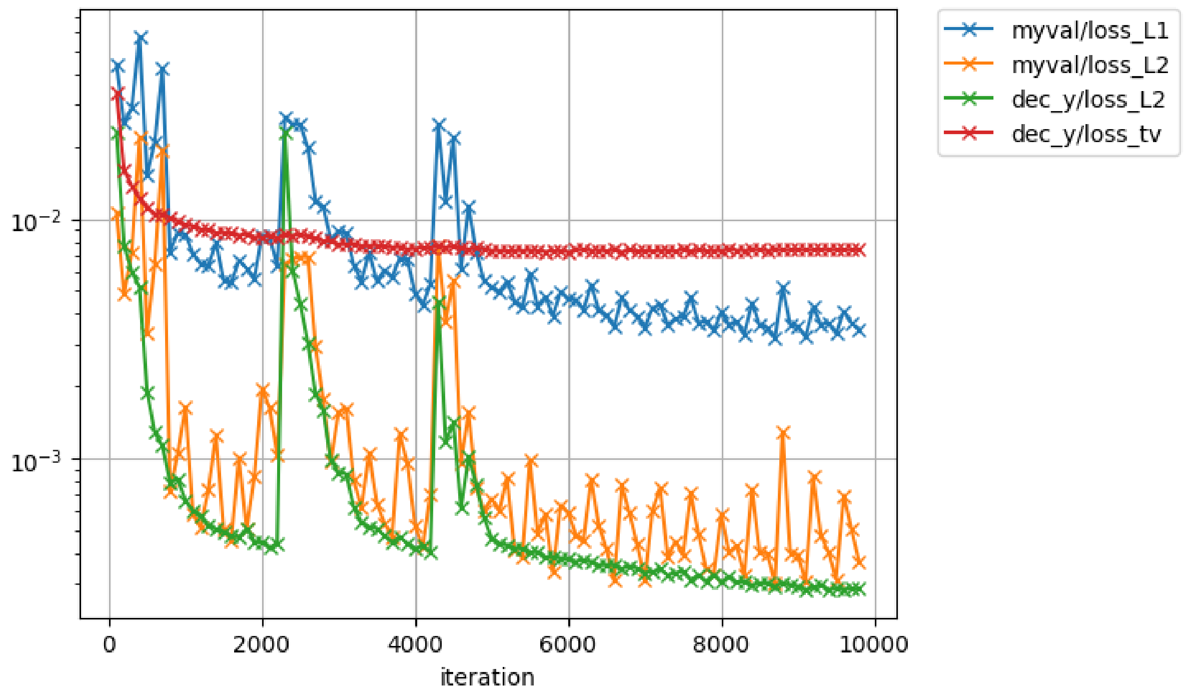

Figure S3. RT2 Generator Loss

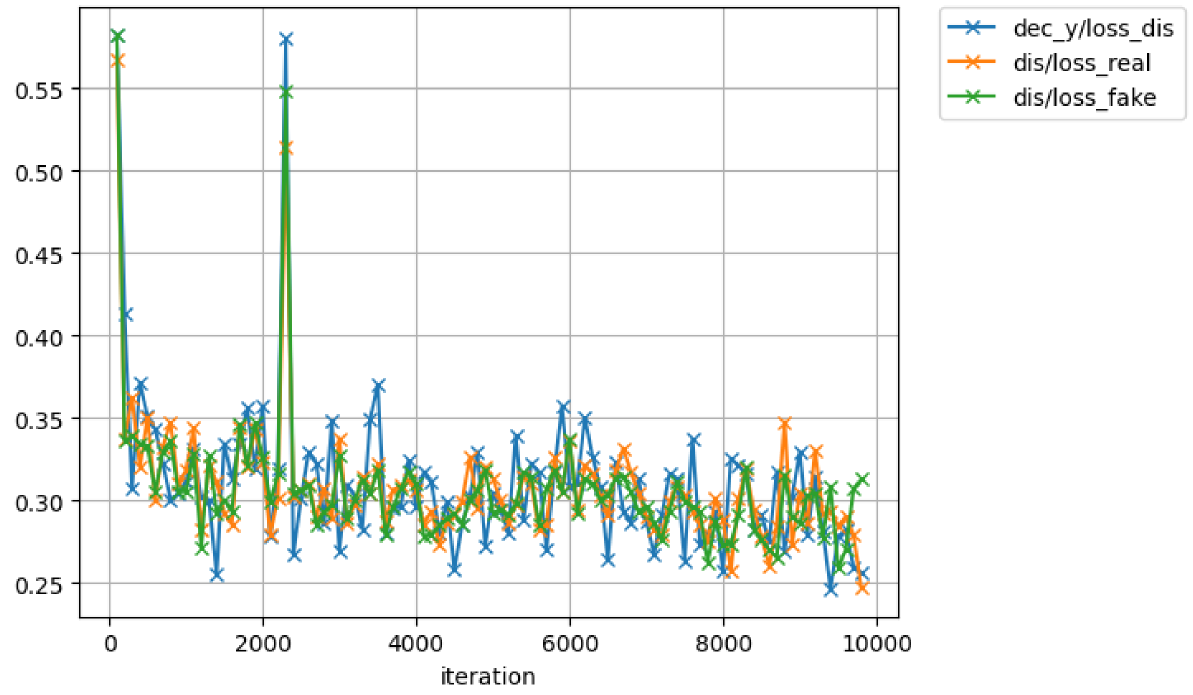

Figure S4. RT2 Discriminator Loss

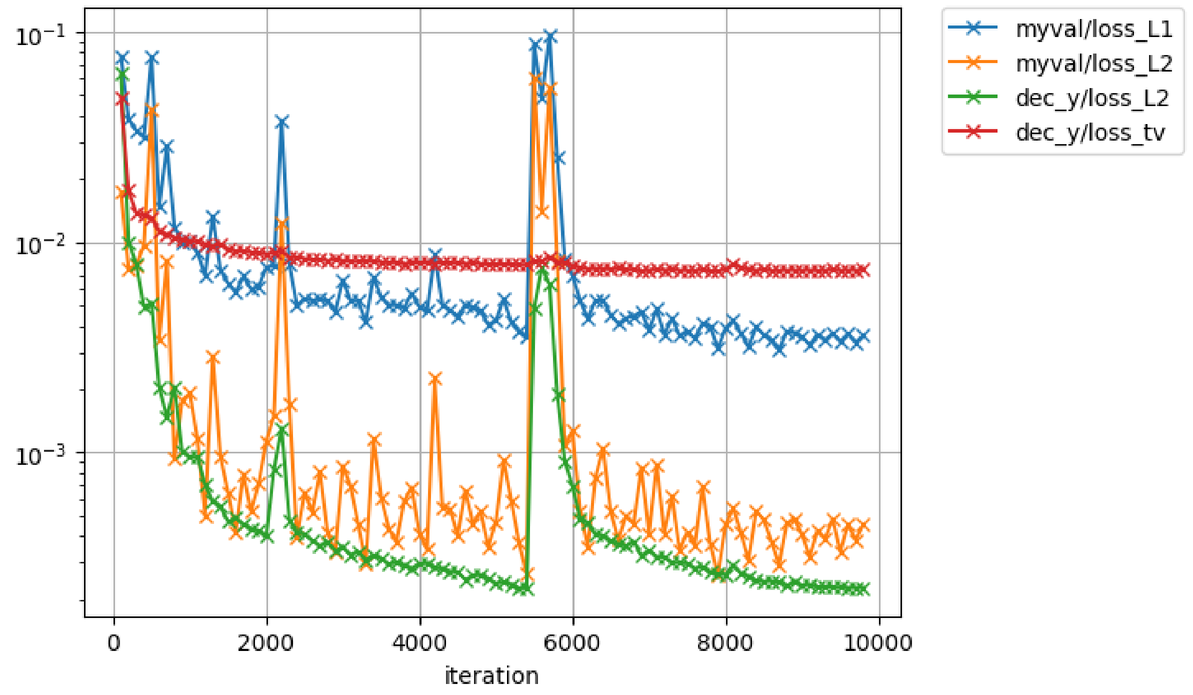

Figure S5. RT0 Generator Loss

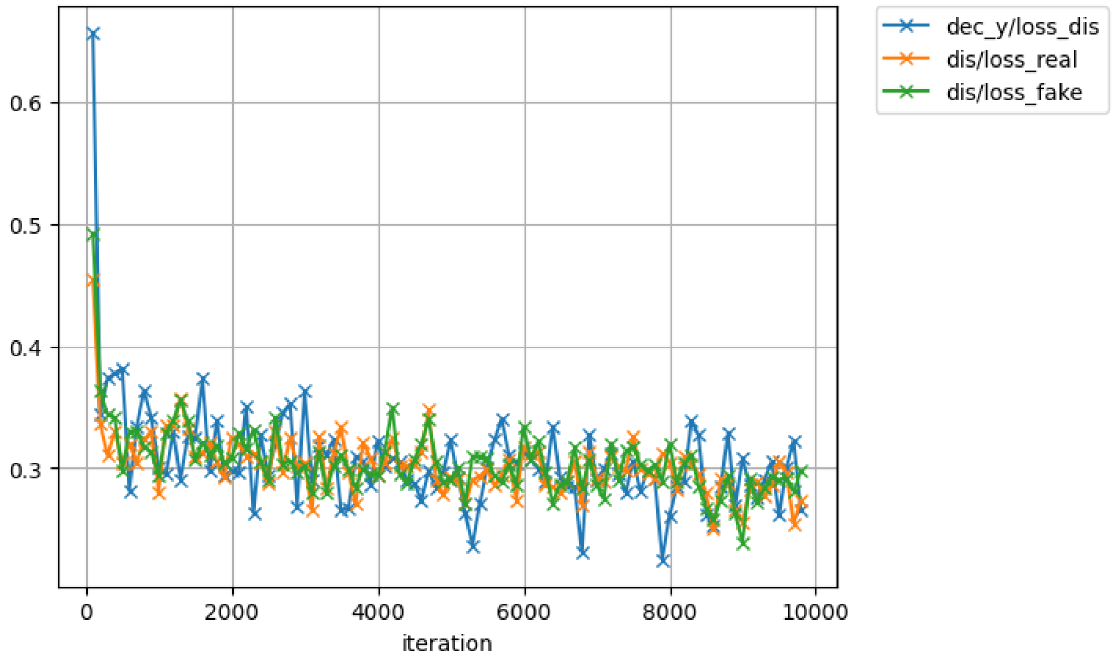

Figure S6. RT0 Discriminator loss

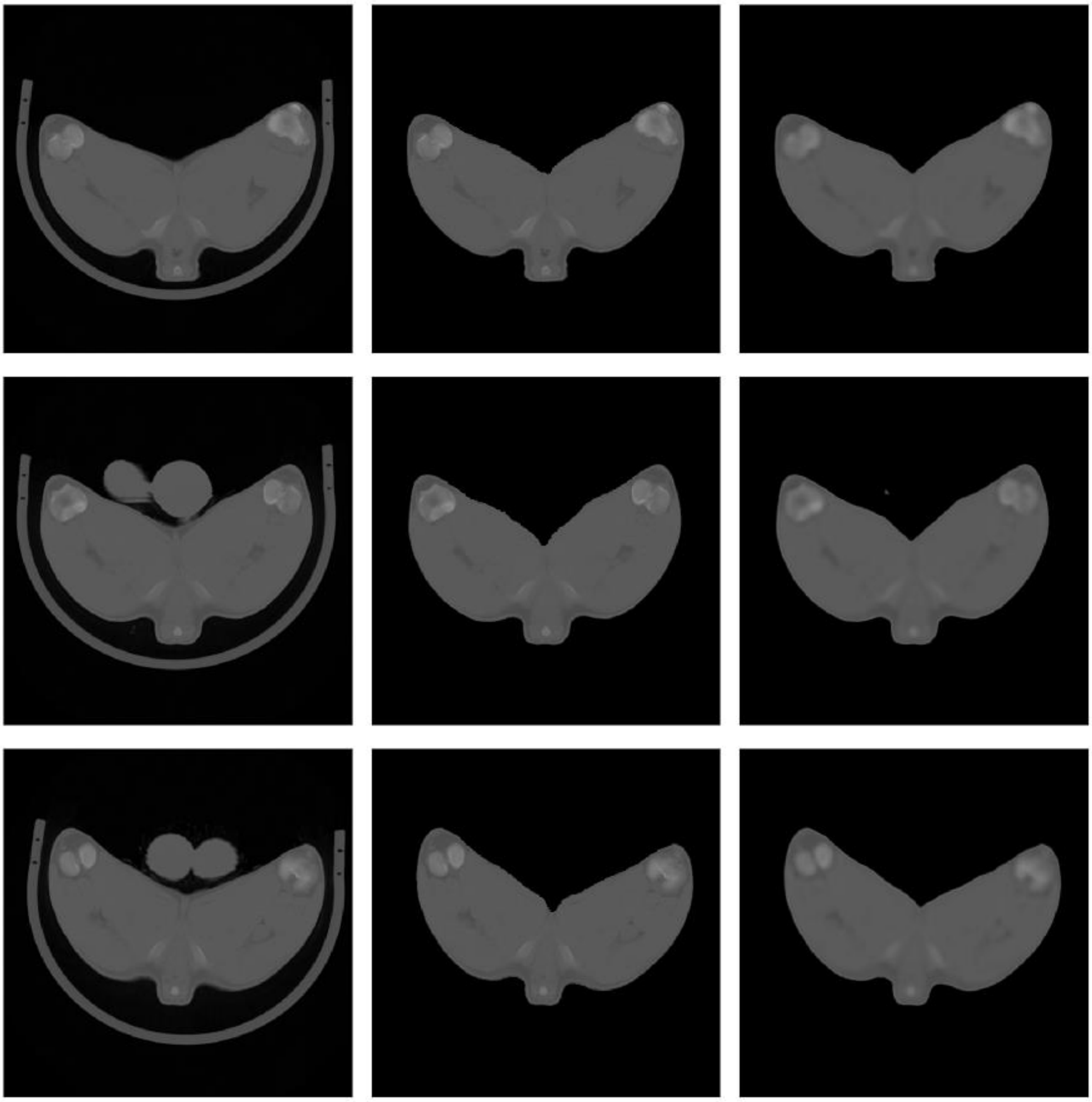

Figure S7. Image quality with RT4.

Raw images (left) were converted into predicted images (right) with RT = 4. Comparison to the ground truth images (middle) show a high degree of blurring. Specifically a reduction in definition between bone:muscle interface was of concern which would impact downstream analysis of bone centre detection.

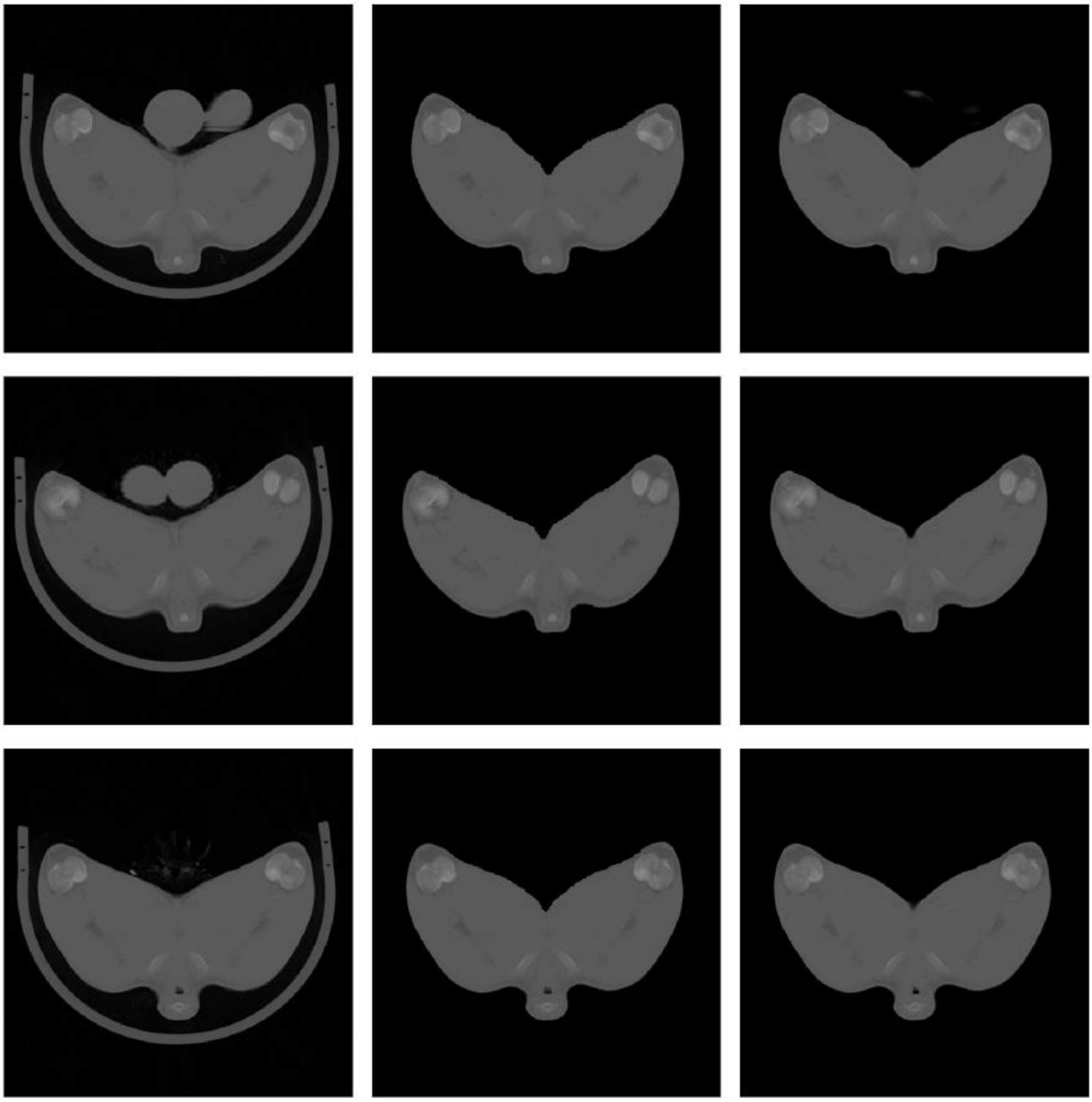

Figure S8. Image quality with RT2.

Raw images (left) were converted into predicted images (right) with RT = 2. Comparison to the ground truth images (middle) show a reduced degree of blurring when compared to images produced when RT=4. With RT=2 features in the bones could be visualised by eye and the tissue boundaries were more distinct.

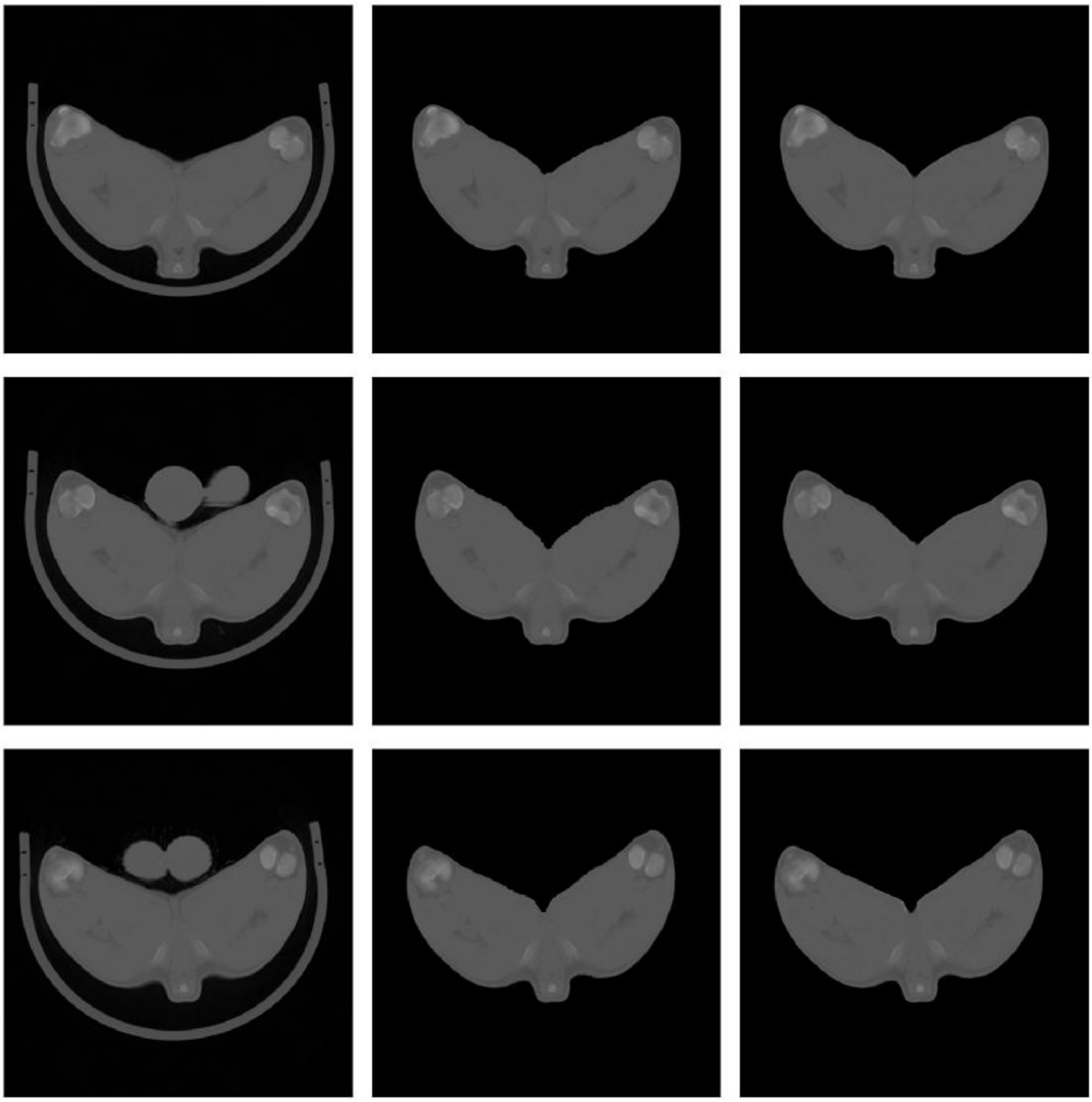

Figure S9. Image quality with RT0.

Raw images (left) were converted into predicted images (right) with RT = 0. Visual comparison to other RT levels showed that RT=0 produced the crispest images, most similar to that of the ground truth. With RT=0 features in the bones could be visualised by eye and the tissue boundaries were more distinct.
