# Supplementary figures and images for: Automated Processing and Phenotype Extraction of Ovine Medical Images Using a Combined Generative Adversarial Network and Computer Vision Pipeline
Source: Sensors (Basel). 2021 Oct 31;21(21):7268. doi: 10.3390/s21217268 (PMC8588206; doi:10.3390/s21217268)

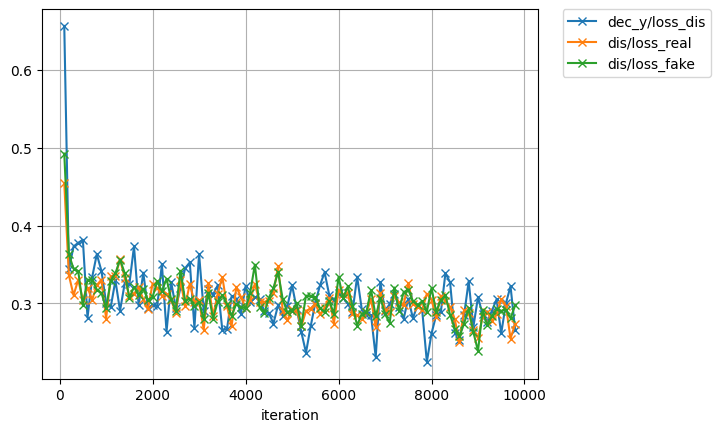

Supplement: Supplementary file 1 [file sensors-21-07268-s001.zip › BothSupplementary/Supplementary Figures/RT0_loss_dis.png]

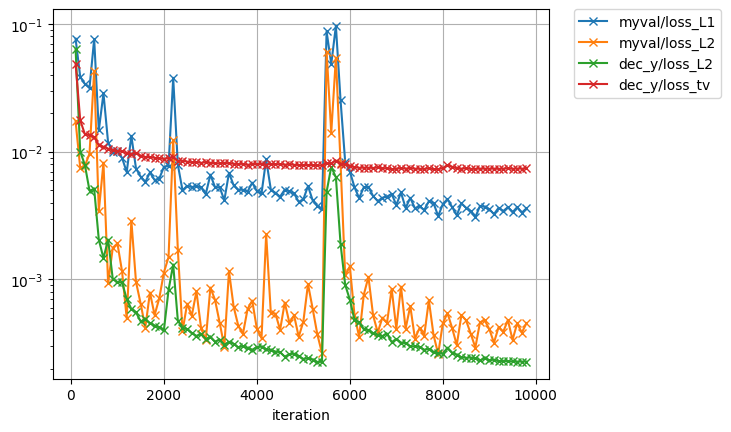

Supplement: Supplementary file 1 [file sensors-21-07268-s001.zip › BothSupplementary/Supplementary Figures/RT0_loss_gen.png]

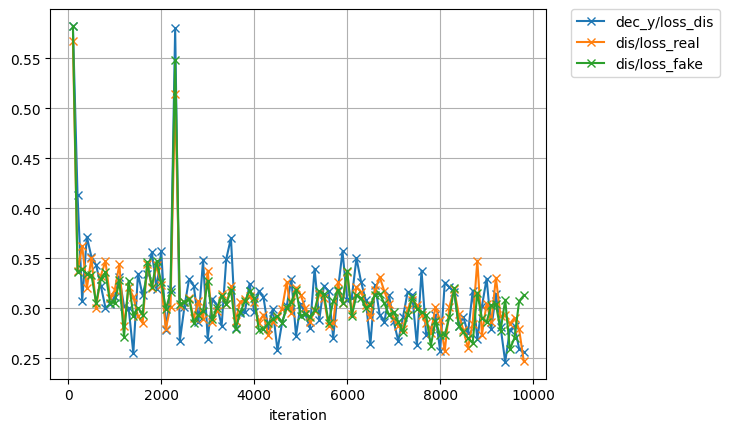

Supplement: Supplementary file 1 [file sensors-21-07268-s001.zip › BothSupplementary/Supplementary Figures/RT2_loss_dis.png]

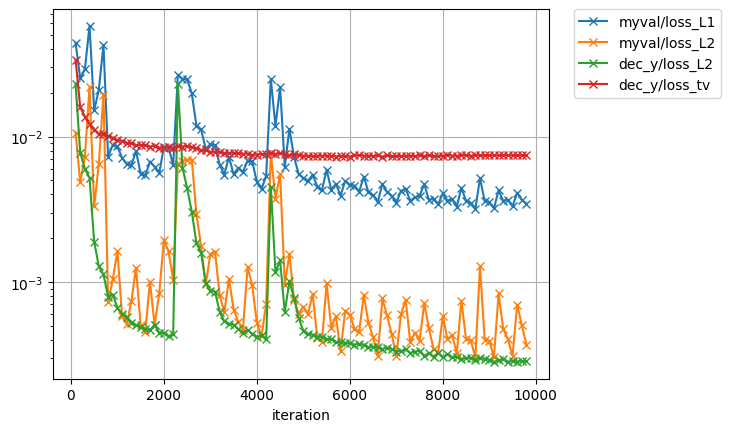

Supplement: Supplementary file 1 [file sensors-21-07268-s001.zip › BothSupplementary/Supplementary Figures/RT2_loss_gen.png]

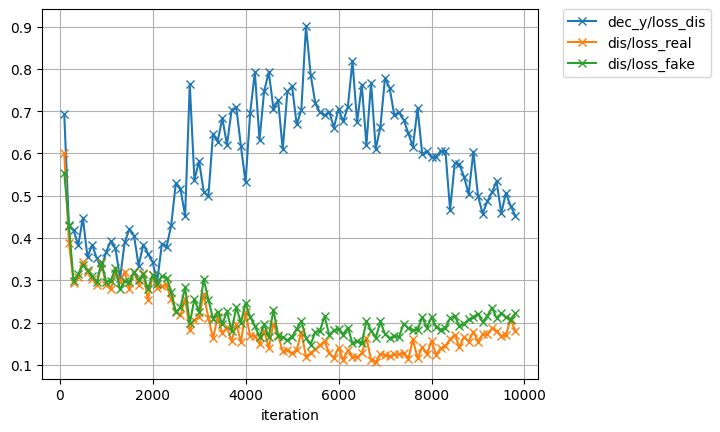

Supplement: Supplementary file 1 [file sensors-21-07268-s001.zip › BothSupplementary/Supplementary Figures/RT4_loss_dis.png]

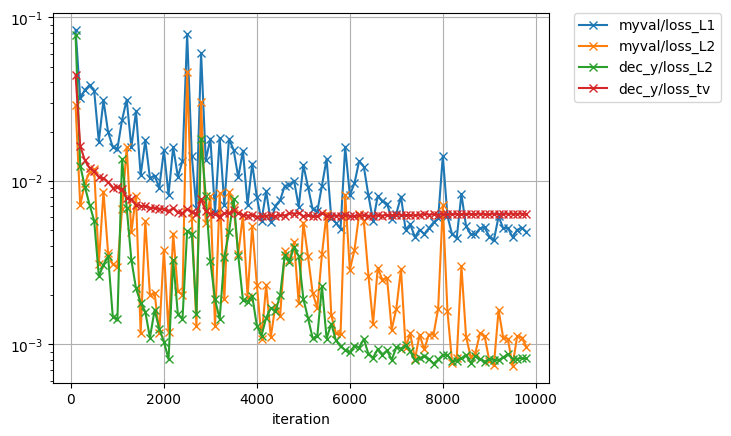

Supplement: Supplementary file 1 [file sensors-21-07268-s001.zip › BothSupplementary/Supplementary Figures/RT4_loss_gen.png]
